# Socioeconomic Predictors of Cognition in Ugandan Children: Implications for Community Interventions

**DOI:** 10.1371/journal.pone.0007898

**Published:** 2009-11-19

**Authors:** Paul Bangirana, Chandy C. John, Richard Idro, Robert O. Opoka, Justus Byarugaba, Anne M. Jurek, Michael J. Boivin

**Affiliations:** 1 Department of Psychiatry, Makerere University School of Medicine, Kampala, Uganda; 2 Department of Public Health Sciences, Karolinska Institutet, Stockholm, Sweden; 3 Department of Pediatrics, University of Minnesota Medical School, Minneapolis, Minnesota, United States of America; 4 Department of Paediatrics and Child Health, Mulago Hospital/Makerere University School of Medicine, Kampala, Uganda; 5 International Neurologic and Psychiatric Epidemiology Program (INPEP), Michigan State University, East Lansing, Michigan, United States of America; 6 Neuropsychology Section, Department of Psychiatry, University of Michigan, Ann Arbor, Michigan, United States of America; University of East Piedmont, Italy

## Abstract

**Background:**

Several interventions to improve cognition in at risk children have been suggested. Identification of key variables predicting cognition is necessary to guide these interventions. This study was conducted to identify these variables in Ugandan children and guide such interventions.

**Methods:**

A cohort of 89 healthy children (45 females) aged 5 to 12 years old were followed over 24 months and had cognitive tests measuring visual spatial processing, memory, attention and spatial learning administered at baseline, 6 months and 24 months. Nutritional status, child's educational level, maternal education, socioeconomic status and quality of the home environment were also measured at baseline. A multivariate, longitudinal model was then used to identify predictors of cognition over the 24 months.

**Results:**

A higher child's education level was associated with better memory (*p* = 0.03), attention (*p* = 0.005) and spatial learning scores over the 24 months (*p* = 0.05); higher nutrition scores predicted better visual spatial processing (*p* = 0.002) and spatial learning scores (*p* = 0.008); and a higher home environment score predicted a better memory score (*p* = 0.03).

**Conclusion:**

Cognition in Ugandan children is predicted by child's education, nutritional status and the home environment. Community interventions to improve cognition may be effective if they target multiple socioeconomic variables.

## Introduction

Children in low income countries are exposed to several diseases and adverse conditions that affect brain development and cognition either through direct injury to the brain or lack of stimulating conditions [1], [2]. Recent estimates put the number of children under five years in low income countries who fail to reach their full cognitive potential because of poverty, poor health and nutrition and deficient care at over 200 million [3]. In light of the above, interventions targeting the environment in which these children live have been suggested [2], [4], [5]. Examples of these interventions are improving child nutrition, early child education, providing a stimulating environment, parenting training and adult education [6], [7], [8], [9], [10].

However, before their implementation, identification of key variables within the child's environment that affect cognition is necessary so as to have focused and effective interventions. Earlier studies have identified nutritional resources, physical development, duration of schooling, parental education, parental occupation, family income, quality of the home environment indicators (e.g. parental interaction, provision of stimulation) and early education enrichment as affecting cognition in African children [8], [9], [10], [11], [12], [13], [14], [15], [16], [17]. These variables differ in the way in which they influence cognition and are thus categorised into proximal and distal variables [18]. Proximal variables are those that the child experiences directly like parental interaction and nutrition while distal variables are those that are experienced indirectly such as family income and parental education [19]. Proximal variables have been reported to influence cognition more than distal variables [13], [19].

The above-mentioned studies with African children either did not look at all these variables at the same time or were cross sectional making it difficult to identify predictors of cognition over time [8], [9], [10], [11], [12], [13], [14], [15], [16]. An exception is a study on Kenyan children's cognitive abilities in which the above predictors and cognition were both measured over three time points [17]. However, the combination of the two cognitive scores into one composite score in this Kenyan study makes it difficult to identify which variables are predictive of specific cognitive abilities. This is especially important since new evidence shows African children perform differently on dynamic and static assessments of cognition [20]. Static assessment is the measurement of pre-existing cognitive skills while the emphasis of dynamic testing is the psychological processes involved in learning and change [20]. These assessments therefore measure different abilities which may be affected by different socioeconomic factors.

Assessment of different cognitive abilities using both dynamic and static tests, rather than a single cognitive score, is therefore necessary to determine which socioeconomic factors predict different cognitive abilities. We present results of a study of healthy Ugandan children who were followed up for 24 months and had different cognitive abilities tested at different points. In addition, both proximal and distal variables including parental education, nutritional status, child's education, socioeconomic indicators and quality of the home environment were measured.

## Methods

### Study Population and Recruitment

The present study was conducted at Mulago Hospital, Kampala, Uganda. Participants were children aged 5 to 12 years recruited as healthy community controls for children with cerebral malaria and uncomplicated malaria taking part in prospective studies looking at the cognitive sequelae of cerebral malaria [21], [22]. They were recruited from the homes or neighbourhoods of children with cerebral malaria and uncomplicated malaria. All children had a medical history and physical examination done to ensure they were healthy at the time of recruitment. Children with a positive smear for malaria were treated with chloroquine and sulfadoxine/pyrimethamine (the first line antimalarial treatment at that time) while those with intestinal parasites were given appropriate antihelminthic medication as per the national health guidelines.

Inclusion criteria were age 5–12 years with no acute illness and signed informed consent from the parent/guardian. Exclusion criteria were (1) a history of meningitis, encephalitis, or any brain disorder, including cerebral malaria; (2) a history of developmental delay; (3) prior admission to the hospital for malnutrition; (4) a history of chronic illness; (5) treatment for an acute illness during the preceding month and (6) admission for malaria during the preceding 6 months.

Ethical approval for the study was granted by the Institutional Review Boards for Human Studies at Makerere University Faculty of Medicine, University Hospitals of Cleveland, Case Western Reserve University, Indiana Wesleyan University, University of Minnesota and the Uganda National Council for Science and Technology.

### Cognitive Assessments

Cognitive testing was done at baseline after physical examination with follow up testing at 6 months and 24 months by testers trained in the administration of the tests. Tests instructions from the test manuals were administered in the local language for children who did not understand English. Instructions were repeated when necessary in cases where the children failed to understand them. In some instances where the child still had difficultly comprehending, the mother or caretaker was asked to explain to the child. Visual spatial processing and memory were measured by the Kaufmann Assessment Battery for Children (KABC) [23] while spatial learning and attention were measured by the Tactual Performance Test (TPT) [24] and the Test of Variables of Attention (TOVA) [25] respectively. These tests have been validated in previous studies with children in Africa and South East Asia [12], [14], [16].

The two scales of the KABC that were administered were the Sequential Processing Scale where problems are solved by arranging the input in sequential order and the Simultaneous Processing Scale where problems are spatial, analogic or organisational and are solved by integrating the input simultaneously [23].

The TPT was administered to the blindfolded child who was required to place six wooden blocks into corresponding holes in a board. The child was first given the blocks to feel their shapes, then feel the holes in the board and their location. The child was given three trials lasting ten minutes each to place the blocks into the holes, the first trial was with the preferred hand, then the non preferred hand and finally with both hands.

The TOVA was administered on a laptop where the child was asked to press a switch whenever the target stimulus (a small black box in the top position) appeared and not to press when the non target stimulus (a small black box in the bottom position) appeared. Outcome scores are inattention (failure to respond), commission (responding to non target), response time (time to respond to target), response time variability (variance in response times) and d' prime (measure of response sensitivity).

Visual spatial processing scores were derived from the Simultaneous Processing Scale of the KABC which comprises of Face Recognition, Gestalt Closure, Triangles, Matrix Analogies, Spatial Memory and Photo Series subscales while memory scores were derived from the Sequential Processing Scale which comprises of Hand Movements, Number Recall and Word Order subscales. Spatial learning was measured by the average time per block for the three trials on the TPT while attention was measured by the d prime score of the TOVA which is one's ability to discriminate between the target and non target stimuli.

### Assessment of Socioeconomic Variables

While the child was doing the baseline cognitive tests, the parent/caregiver was asked about the quality of child's home environment. The quality of the home environment was measured by the Middle Childhood Home Observation for the Measurement of the Environment (MC-HOME) [26]. The MC-HOME is used to assess the stimulation and learning opportunities offered by the child's home environment. Studies using similar home evaluations have shown that the child's home environment affects its cognitive development [13], [27]. The MC-HOME consists of eight subscales; Responsivity, Encouragement of maturity, Emotional climate, Learning materials and opportunities, Enrichment, Family companionship, Family integration and Physical environment. It has 59 items however item 40 ‘Family member has taken child to (or arranged for child to visit) a scientific, historical or art museum within past year’ was omitted because it was deemed not applicable to most of the children in the sample thus leaving 58 items for use in the study. This modified MC-HOME had an inter-item reliability of 0.85.

Nutrition was assessed as in our previous studies [21], [22] by comparing weight for age to published norms [28] and obtaining a standardized z-score (Statistical Analysis System (SAS) release 9.1, SAS Institute, Inc., Cary, North Carolina). Socioeconomic status was assessed using a scoring instrument incorporating a checklist of material possessions, house structure, living density, food resources and access to electricity and clean water. Level of education of the child and mother were scored as follows: None = 0, Nursery = 1, Primary school grades 1−7 = 2−8, Secondary education = 9, Post-secondary school = 10.

Children spend one to three years in nursery school (pre-primary) and seven years in primary school for classes Primary one to Primary seven (P1 to P7). The age of entry into nursery and primary varies because parents may delay to take children to school for various reasons. The Uganda government has a Universal Primary Education policy where all children are entitled to free primary education where schools are urged to promote children to the next class regardless of the performance.

Six socioeconomic variables were obtained from the above assessments; quality of the home environment (MC-HOME score), nutritional status, maternal education level, child's education level and socioeconomic status (SES) score.

### Statistical Analysis

Data were entered into databases using FileMaker Pro 7 and analysed using Statistical Package for the Social Sciences (SPSS) version 11.0 and SAS 9.1. Raw cognitive test scores were log transformed to generate normal distributions, with a higher score for visual spatial processing, memory and attention reflecting a better score and a lower score for spatial learning reflecting a better score. Pearson's correlations were run between test scores at baseline and 6 months and between 6 months scores and 24 months scores to determine the test-retest reliabilities of the tests. Similar correlations were also run between the socioeconomic factors to determine the relationships between them. A longitudinal mixed effects model [29] was used to study the effects of socioeconomic factors and other covariates (baseline age, gender, weight-for-age z-score, child's education level, home score, social economic status (SES), and maternal education) on cognitive assessments, since the same cognitive assessments were performed at three time points. In the regression analyses, the predictor variable coefficients were calculated for each of the four outcome variables (log-transformed scores in the areas of visual spatial processing, learning, attention and working memory). Exponentiated coefficients represent the percent change in geometric mean per unit on the non-transformed scale of the predictor variable [30].

## Results

### Demographic Characteristics

Eighty-nine children were recruited at baseline of which 87 were followed up at 6 months (a male and female were lost at 6 months) and 79 at 24 months (3 males and 5 females were lost at 24 months). There was a similar proportion of males to females at baseline (45 females and 44 males), and the mean age of enrolled children was 7.92 (standard deviation [SD] 2.04). The mean level of education was 3.02 (SD 1.78) for the children corresponding to primary four, and 6.18 (SD 2.12) for the mothers corresponding to primary seven (Table 1). All children had normal blood counts including haemoglobin level (data not shown).

**Table 1:** Demographic characteristics of study participants at baseline.

Domain	Children with available data	%	Mean	SD	Possible range (min-max)
Age, years/months	89		7.92	2.04	5–12
5 years	17	19.1			
6 years	17	19.1			
7 years	15	16.9			
8 years	13	14.6			
9 years	9	10.1			
10 years	7	7.9			
11 years	9	10.1			
12 years	2	2.2			
Level of education (Child)	89		3.02	1.78	0–8
Level of education (Mother)	81		6.18	2.12	0–9
Weight for age z score (WAZ)	87		−1.07	1.10	−3.95–1.70
MC-HOME score	86		29.60	7.00	10–43
Socioeconomic status (SES) score	84		10.27	2.85	6–18

MC-HOME, Middle Childhood Home Observation for the Measurement of the Environment; SD, Standard deviation.

### Correlations between Socioeconomic Variables

Pearson's correlations were run between the socioeconomic variables to identify the interrelationships between them. Socioeconomic status correlated with MC-HOME score (r = 0.37), maternal education (r = 0.22) and child's education (r = 0.26). Maternal education correlated with MC-HOME score (r = 0.32) and child's education correlated with MC-HOME score (r = 0.26). Table 2. The cognitive tests were relatively stable over the 24 months study period with test-retest reliabilities ranging from 0.55 to 0.84. Table 3.

**Table 2:** Correlations between socioeconomic variables.

Variable	1	2	3	4	5
1 SES score	-	**0.37**	**0.22**	0.05	**0.26**
2 MC-HOME Score		-	**0.32**	0.08	**0.26**
3 Level of education (Mother)			-	−0.12	0.20
4 Weight for age z score (WAZ)				-	0.14
5 Level of education (Child)					-

SES, Social Economic Status; MC-HOME, Middle Childhood Home Observation for the Measurement of the Environment.

**Table 3:** Descriptive statistics and test-retest reliabilities of the cognitive scores.

	Descriptive statistics^1^								Correlations	
Cognitive domain	Mean	SD	Lowest	Highest	Range	Median	Skewness	Kurtosis	0 to 6 months	6 to 24 months
Working memory	3.19	0.35	2.20	3.95	1.75	3.22	−0.54	0.13	0.84	0.81
Visual spatial processing	3.26	1.21	−2.30	4.41	6.71	3.47	−3.29	13.16	0.67	0.82
Spatial learning	3.75	0.93	1.84	6.68	4.85	3.44	1.42	2.07	0.55	0.64
Attention	0.81	0.49	−0.82	1.91	2.73	0.85	−1.13	2.75	0.71	0.69

1 Descriptive statistics for the baseline (0 months) scores only.

### Relationship of Socioeconomic Variables to Cognitive Outcomes

After adjustment for all other variables, higher education level of the child predicted memory (percent change 6% (1.06), 95% confidence interval (CI)  = 1.0 to 10.0, *p* = 0.03), attention (percent change 12% (1.12), 95% CI = 3.0 to 19.0, *p* = 0.005) and spatial learning scores over the 24 months (percent change −11% (0.89), 95% CI = −19.0 to −0.2, *p* = 0.05; negative percent change better for spatial learning) (Table 4). Better nutrition (higher weight for age z-score) predicted visual spatial processing and spatial learning scores (percent change 13% (1.13), 95% CI = 4.0 to 20.0, *p* = 0.002 and percent change −10% (0.90), 95% CI = −19.0 to −3.0, *p* = 0.008 respectively), and a higher home environment score predicted better memory (percent change 1% (1.01), 95% CI = 0.1 to 2.0, *p* = 0.03).

**Table 4:** Exponentiated coefficients represent percent change in the geometric mean per unit of the predictor variable^1^.

Predictor variables' coefficients					
Cognitive domains	SES score	MC-HOME score	Level of education (Mother)	WAZ	Level of education (Child)
Visual spatial processing	1.02	1.01	1.00	1.13**	1.08
Working memory	1.01	**1.01** *	1.01	1.04	**1.06** *
Attention	1.00	1.01	1.01	0.99	1.12**
Spatial learning	1.02	0.99	0.99	0.90**	**0.91** *

*p<0.05; **p<0.01; SES, Social Economic Status; MC-HOME, Middle Childhood Home Observation for the Measurement of the Environment; WAZ, weight for age Z score.

1 Exponentiated values represent the percent increase or decrease in the geometric mean on the non-transformed scale. Values greater than 1 provide a percent increase in the geometric mean per unit of the predictor variable; values less than 1 are a percent decrease in the geometric mean per unit of the predictor variable (subtract from 1). For visual spatial processing, attention and working memory, exponentiated coefficients greater than 1 reflect a better outcome with increased predictor value; for spatial learning, exponentiated coefficients less than 1 reflect a better outcome with increased predictor value.

## Discussion

This study prospectively examined cognition in healthy Ugandan children providing a unique opportunity to identify predictors of cognition in this group over a 24-month period. In the longitudinal model, the child's level of education, nutritional status and home environment were the most important predictors of cognitive test scores over the 24 months with child's education level predicting memory, attention and spatial learning, nutritional status predicting visual spatial processing and spatial learning while quality of the home environment predicted memory. Maternal education and SES did not predict any cognitive outcome.

Higher education level of a child predicted improved cognitive outcome in three of the four cognitive areas tested. The benefits of a child's education were also seen in an earlier study of Congolese children where child's education was associated with better memory performance [15]. Children with higher education performed better than their colleagues of lower education on the KABC's sequential processing. These findings are not surprising, but are perhaps reassuring, since the goal of education is to improve general cognition. Education helps people understand general rules, apply cognitive skills to new situations and also strengthens memory and thinking skills probably through the continuous need to remember class work and solve problems based on past learning [31]. Schooling may also influence performance on cognitive tests through the stimulation the child gets from the school environment since enriched environments with more stimulation are associated with better cognitive outcomes [8]. It may also make them test-wise and perform better on cognitive tests especially those developed in the west.

The association of better nutritional status with improved visual spatial processing and spatial learning is consistent with those from earlier studies done among Kenyan, Congolese and Lao children where anthropometric indicators of nutritional status (weight, height and arm circumference in proportion to height and head circumference) predicted spatial learning and psychomotor scores [11], [14], [16] but did not predict memory performance [11], [14], [16]. Children with better nutritional status also had better visual spatial skills, a finding consistent with nutritional intervention studies in Kenya where children receiving a diet with meat supplementation performed better than those with a milk diet, energy diet or no supplementation on the Raven's Coloured Progressive Matrices [6], [7]. Raven's matrices measure the child's ability to reason by analogy, organise perceptual detail and form comparison [6], skills that are also measured by the KABC's simultaneous processing scale, which was our study's visual spatial measure. Meat intake was associated with greater increase in middle upper arm muscle area (a measure of lean body mass) in Kenyan children [7] suggesting that the improved scores in visual spatial skills in this study might be mediated by improved nutrition. Nutrition's effects on the brain have direct implications for the child's cognitive skills by providing the brain with necessary nutrients during its development that enhance its development and function [32]. The importance of nutrition in promoting the child's cognition has been noted in many other studies, including a review by Walker et al in which three of the four key risk factors for poor cognitive development in children were related to nutrition (stunting, iodine deficiency and iron deficiency anaemia) [1].

Nutrition appeared to specifically affect visual spatial learning and spatial learning but not attention or working memory in our study. Visual spatial learning and spatial learning are both dynamic measures of cognitive ability whose principal outcome measure is a child's improvement in performance from one session to the next in learning a complex cognitive task while attention and working memory are static measures that capture state-dependent aspects of cognitive ability as one-time assessments, and do so in the absence of learning improvements across sessions [33]. Dynamic assessments are more sensitive to the long term effects of poverty related developmental impairment than static measures and are thus the best way to reveal the long-term impact of poverty on children in low income countries [20]. In this respect, the KABC's Simultaneous Processing measure of visual spatial processing and TPT's measure of visual spatial learning may be inherently more sensitive to quality of developmental milieu, as impacted upon by, for example, long-term nutritional status than TOVA's attention and KABC's Sequential Processing measure of working memory which are more sensitive to acute brain injury infections [20], [21], [22]. This could explain why nutrition only predicted spatial learning and visual spatial processing and not attention or working memory.

The amount of stimulation in the home environment predicted memory in the children. The kind of short-term memory measured by the KABC in our study is working memory which has parietal lobe, frontal lobe and hippocampal involvement [34], [35]. Experimental studies in animals show more neurons produced in the hippocampus and increased dendritic length in the parietal lobe and as a result of environmental enrichment which may partly explain the effect of the home environment on memory [36], [37], [38]. The quality of the home environment is also dependent on maternal education [13] and there was a correlation between MC-HOME scores and maternal education in our study. In an earlier study in Lao children, maternal education predicted memory as measured by the KABC [16]. These results should be interpreted with caution given the small coefficients for the MC-HOME.

A strength of the present study is the inclusion of maternal education, home environment, and socioeconomic status and other important variables into a multivariate analysis. This analysis demonstrated that among MC-HOME score, maternal education and socioeconomic status, only MC-HOME score independently predicted a cognitive outcome. Our findings are consistent with previous studies which have documented that the quality of the home environment is a better predictor of cognition than socioeconomic indicators like household income and parental education [13], [39]. A recent study in Kenya also showed no direct relationship between SES (measured by mother's education and household wealth) and psychomotor development [11].

Our study findings suggest that proximal variables (child's education, nutritional status and home environment) are better predictors of cognition than distal variables (parental education and socioeconomic status) and this is because the former have a direct effect on the child's cognitive development than the latter. This however does not mean that distal variables are unimportant since SES has been associated with availability of a stimulating environment and maternal education is important for the provision of a good stimulating environment and better nutrition [15], [40], [41]. A high SES may also be needed to keep the child in school by providing fees or getting better education.

The multivariate analysis demonstrated that maternal education and SES are not associated with a significant difference in cognitive outcomes after adjusting for home environment, nutrition and education.

These findings not only point out the need for interventions that affect proximal and distal variables in at-risk groups of children, but also demonstrate the importance of assessment of these variables in studies of cognition, as they may be important confounding variables. Strengths of the present study included the detailed evaluation of cognition, repeated cognitive testing over time, and careful testing of socioeconomic variables. Limitations of the study included: testing socioeconomic variables at baseline only, lack of a battery to test language skills, the lack of testing children under 5 years of age (excluded because the cognitive tests used have not been validated in younger children), a wide age range of 5 to 12 years and a lack of assessment of detailed nutritional characteristics, such as levels of iron and other micronutrients.

In conclusion, specific cognitive outcomes in healthy Ugandan children are predicted by the child's education, nutrition and home environment. These factors should be considered as confounders when studies of cognition are conducted in Uganda or other sub-Saharan African countries. Though these three variables are better predictors of cognition, effective community interventions need to target all key socioeconomic variables, since many other variables such as maternal education and socioeconomic status also affect a child's education, nutrition and home environment.
